# The Interplay of Reovirus with Autophagy

**DOI:** 10.1155/2014/483657

**Published:** 2014-03-10

**Authors:** Hung-Chuan Chiu, Sarah Richart, Fong-Yuan Lin, Wei-Li Hsu, Hung-Jen Liu

**Affiliations:** ^1^Institute of Molecular Biology, National Chung Hsing University, Taichung 402, Taiwan; ^2^Department of Biology and Chemistry, Azusa Pacific University, Azusa, CA 91702, USA; ^3^Graduate Institute of Microbiology and Public Health, National Chung Hsing University, Taichung 402, Taiwan; ^4^Agricultural Biotechnology Center, National Chung Hsing University, Taichung 402, Taiwan; ^5^Rong Hsing Research Center for Translational Medicine, National Chung Hsing University, Taichung 402, Taiwan

## Abstract

Autophagy participates in multiple fundamental physiological processes, including survival, differentiation, development, and cellular homeostasis. It eliminates cytoplasmic protein aggregates and damaged organelles by triggering a series of events: sequestering the protein substrates into double-membrane vesicles, fusing the vesicles with lysosomes, and then degrading the autophagic contents. This degradation pathway is also involved in various disorders, for instance, cancers and infectious diseases. This paper provides an overview of modulation of autophagy in the course of reovirus infection and also the interplay of autophagy and reovirus.

## 1. Introduction 

**Figure 1:**
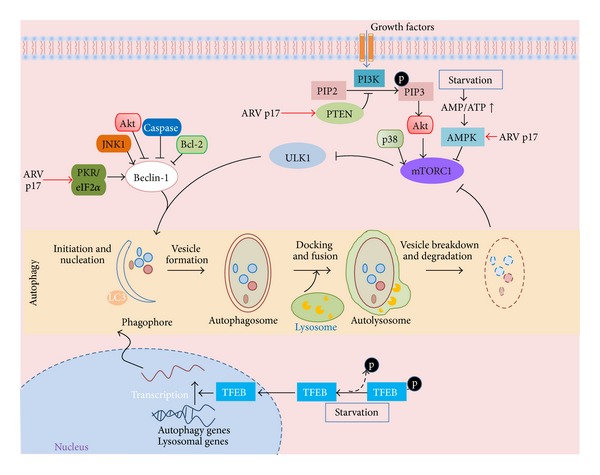
Regulation of autophagy by ARV. The autophagy process contains several distinct steps, including membrane isolation, nucleation, vesicle formation, fusion of autophagosome with lysosome, and degradation of the cargo followed by release of the degradation products back into the cytosol. The activity of autophagy is regulated by several cellular signaling pathways; mTOR-dependent pathway (via mTORC1), and mTOR-independent pathways (such as TFEB-mediated pathway and cyclical Ca^2+^-calpain-G*α*s and cAMP-Epac-PLC-*ε*-IP3 pathways). Starvation inhibits mTORC1, a downregulator of autophagy, and also induces dephosphorylation of TFEB resulting in the activation of autophagy [2]. Infection of ARV promotes autophagy via the action of p17 protein at the various steps of PI3K-Akt-mTORC1 pathway and also on activation of PKR/eIF2*α* signaling [9]. In the case of Beclin-1 regulation, Wirawan et al. demonstrated that caspase activation in cells undergoing autophagy has been demonstrated to cleave Beclin-1, thereby inducing apoptosis [10]. The antiapoptotic protein, Bcl-2, interacts with Beclin-1 and inhibits Beclin 1-dependent autophagy [11]. Autophagy and apoptosis are basic cellular pathways that are regulated by JNK-mediated Bcl-2 phosphorylation [12]. JNK1-mediated Bcl-2 phosphorylation interferes with its binding to Beclin-1, thereby promoting autophagy induction [12]. More recently, a report by Wang et al. demonstrated that Akt-mediated phosphorylation of Beclin-1 increased its interactions with 14-3-3 and vimentin, leading to autophagy inhibition [13].

Autophagy is a cellular degradation process by which cytoplasmic substrates are processed for lysosomal degradation. In eukaryotic cells, two types of autophagy are involved in dynamic rearrangement of the sequestering membrane, namely, microautophagy and macroautophagy. Microautophagy involves the engulfment of cytoplasm directly at the surface of lysosome by invagination, protrusion, and septation of the lysosomal limiting membrane [1]. In this review, we focus on macroautophagy, hereafter referred to as autophagy. In general, the process of autophagy begins by double-membrane initiation, followed by cargo recognition, and fusion with lysosomes (autophagosome maturation) that results in cargo degradation (Figure 1). The membrane of autophagosomes is derived from the plasma membrane and cellular organelles. After cytoplasmic material (the cargo) is sequestered in cup-shaped double-membrane structures (phagophores), the edges of the membranes are then sealed and the formation of autophagosomes is complete. By trafficking along microtubules, autophagosomes migrate to the proximity of lysosomes for fusion. Subsequently, autophagic cargos containing cytosolic constituents are degraded by lysozyme activity [2]. This process will eliminate intracellular pathogens and damaged organelles which are too large for the proteasome, resulting in the recycling of nutrients and the generation of energy [3].

Autophagy is regulated by several cellular signaling pathways, class I phosphoinositide 3-kinase (PI3K)-protein kinase B (PKB)-mammalian target of rapamycin complex 1 (mTORC1), and other mTOR-independent pathways, for instance, cyclical Ca^2+^-calpain-G*α*s and cAMP-Epac-PLC-*ε*-IP3 and the basic helix-loop-helix leucine zipper transcription factor EB (TFEB)-mediated pathway [2] (Figure 1). The biogenesis of autophagosomes requires more than fifteen autophagy-related (Atg) proteins, including class III PI3Ks, UNC51-like kinase 1 (ULK1) complex, and microtubule-associated protein 1 light-chain 3 (LC3) that act on different modules. These Atg proteins are sequentially recruited at the phagophore assembly site. For instance, ULK1 and PI3K complexes (composed of Beclin-1 and class III PI3K etc.) congregate at the phagophore assembly site for initiating autophagy. And the proteins Atg8 and LC3 are involved in the expansion and fusion of phagophore edges and can recruit adaptor proteins to autophagosomes [4].

Autophagy has been considered to be a stress response that allows eukaryotic organisms to survive during harsh conditions, such as nutrient starvation, energy depletion [5], or reactive oxygen stress [6]. Under starvation conditions, the induction of autophagy degrades proteins, carbohydrates, and lipids that allow the cell to maintain energy levels; this adaptation has been shown to be important for survival during neonatal starvation in mice, for example, [7]. In addition, inhibition of autophagy accelerates starvation-induced apoptosis, indicating that autophagy machinery exerts a crucial role under conditions of nutrient deprivation [8].

It has been demonstrated that autophagy is also activated by many pathogens and contributes to host defenses confronting microbial infection. In the past decade, the mechanisms by which viruses either subvert or enhance autophagy in their replication and pathogenesis have been intensively studied [3, 14, 15]. In this review, we describe recent research findings which examine the modulation of autophagy by mammalian reovirus (MRV) and by avian reovirus (ARV), as well as the autophagy signaling modulated by other viral proteins.

Reovirus, named for respiratory, enteric, and orphan, belongs to genus* Orthoreovirus *in family Reoviridaeand is a nonenveloped virus with a genome consisting of 10 double-stranded RNA segments. Reovirus has a wide host range, from reptiles to birds to mammals. In general, reovirus infections in humans are asymptomatic and mostly restricted to the upper respiratory and gastrointestinal tracts [16]. While reovirus is not a significant human pathogen, interestingly, MRV has the ability to preferentially kill a variety of tumor cells (both* in vivo *and* in vitro*) by oncolysis, particularly MRV type 3 (strain Dearing), which has led to its use as a cancer treatment in several different clinical trials, including head and neck tumors [17], and more recently multiple myeloma [18]. In addition, it is clear that apoptosis plays a role in reovirus oncolysis. However, the interplay between autophagy and apoptosis which leads to oncolysis in MRV infection remains largely unknown.

## 2. Viral Induction of Autophagy in Oncolysis

Several lines of evidence have shown that MRV selectively replicates in cells with activated Ras signaling pathways [19–21]. In fact, cells that are resistant to reovirus become susceptible either by the introduction of constitutively activated epidermal growth factor receptor (*v-erbB *oncogene) which activates Ras [20] or by introduction of active Ras [22].* Ras* is a protooncogene that encodes a key regulator of mitogenic signals. Reoviruses, while they can infect a wide variety of cells from various hosts, selectively cause lysis in transformed cells with aberrant signaling pathways and have been demonstrated to target multiple cancers, including multiple myeloma [23–25].

Multiple myeloma (MM) is a neoplasm of plasma cell origin and accounts for ~10% of all blood cell malignancies [26]. Mutations of N-Ras or K-Ras resulting in constitutive activity have been identified in myeloma with a frequency approaching 50% [27, 28]. In addition to activation of Ras pathway, multiple myeloma has been characterized by the activation of several other signaling pathways which promote tumorogenesis, for instance, MEK/ERK, PI3K/Akt, mTOR/p70S6 kinase, and NF-*κ*B pathways [23–25]. These activated pathways may increase the lytic infection efficiency of MRV which suggested that reovirus may be an effective alternative therapy for MM. Oncolytic viruses are very attractive cancer therapies since they replicate and may continue to spread and infect more tumor cells. MRV has been demonstrated to target and kill MM, and a phase I clinical trial has recently been initiated [18, 29]. Thirukkumaran et al. demonstrated that apoptosis induced by MRV infection contributes to oncolysis of human multiple myeloma cell lines (HMCL) [30]. In addition to apoptosis, the formation of cellular autophagosome puncta containing LC3-II, indicating activation of autophagy, was detected in myeloma cells at 24–48 hours after infection of live reovirus and this autophagy activation was further blocked by autophagy inhibitor 3-methyladenine (3-MA) [18].

While the role of autophagy in MRV-induced cell death has not yet been characterized, recent studies of other oncolytic viruses (OV), such as adenovirus and human simplex virus (HSV), may shed some light on this question. Infection of melanoma cells with a HSV type 2 mutant (ΔPK) containing a deletion of the ICP10 gene (encoding a viral protein kinase) reduced the melanoma tumor burden via activation of a number of functionally distinct proteases (caspase-3, caspase-7, and calpain) and also via upregulation of the proapoptotic protein H11/HspB8, as well as Beclin-1 [31], a critical autophagy protein that has been shown to be a potent suppressor of human brain tumours and melanomas [32, 33]. These results demonstrated that the ΔPK virus-induced oncolytic activity is involved in nonredundant virus-induced programmed cell death (PCD) pathways, including apoptosis and autophagy processes.

Adenoviruses (Ads) deficient in E1b function, like reoviruses, can selectively replicate in and cause oncolysis of cancer cells [34]. In a study using a series of Ads, Rodriguez-Rocha et al. demonstrated that both wild-type (Ad5) and E1b-deleted (Adhz60) adenoviruses induce autophagosome formation as evidenced by the enhanced incorporation of LC3-II to autophagosomes and the formation of the Atg12–Atg5 complexes in a human lung cancer cell line (A549) [35]. However, deletion of both E1a and E1b (which abolishes viral replication) failed to activate formation of autophagosomes. 3-MA treatment of cells infected with either Ad5 or Adhz60 not only inhibited autophagy, but also decreased the accumulation of viral proteins as well as the yield of virus progeny. These findings indicate that autophagy induced by adenovirus correlates positively with virus infection. Conversely, Botta et al. proposed that autophagy acts as a cell survival response: despite observing the activation of autophagy in glioma cells infected with dl922-947, a replication competent oncolytic Ad with a 24-bp deletion in E1a conserved region-2, they found that, paradoxically, the main negative regulator of autophagy (the Akt/mTOR/p70s6k pathway) was activated and the positive regulator (the ERK1/2 pathway) was inhibited during Ad viral infection [36]. Moreover, inhibition of autophagic pathways by either 3-MA or chloroquine increased the replication and cytotoxicity of the dl922-947 virus, indicating that autophagy might play a survival and/or defensive role in glioma cells infected by oncolytic Ads with E1a-deficiencies. Since different Ad viruses were tested on different cancer cells in each of these studies, the interplay between autophagy and viral infections is likely complicated; the activation of autophagy and its subsequent effect are most likely dependent on virus and cell type.

## 3. Avian Reovirus Modulates the Host Autophagy Pathway

Recently, Meng and his colleagues demonstrated that ARV S1133 strain induces autophagy in both primary chicken embryonic fibroblast (CEF) cells and mammalian cells (Vero) by showing that virus infection increased the number of double-membrane vesicles, dots of GFP-LC3 (from transient expression), and the elevated production of the autophagy indicator, LC3-II [47]. They also demonstrated that ARV infection triggers autophagy through PI3K/Akt/mTOR pathway [47]. However, the detailed mechanism by which ARV triggers the autophagy pathway was not revealed until recently; Chi et al. demonstrated that ARV nonstructural protein p17 functions as an activator of autophagy [9]. In the presence of ARV protein p17, expression levels of Beclin-1 and LC3-II were increased [9] (Figure 1). Moreover, ARV protein p17 was found to regulate p53/PTEN/mTOR and adenosine monophosphate-activated protein kinase (AMPK) and led to downregulation of the key negative regulator mTOR, which in turn induces autophagy. In addition, ARV also activates the PKR/eIF2*α* pathway that has been shown to promote autophagy [9]. A previous report by Talloczy et al. also suggested that virus induced autophagy by the eIF2 alpha kinase signaling pathway [48]. Furthermore, this team discovered that ARV triggered AMPK signaling facilitates activation of the MKK 3/6 and MAPK p38 signaling pathway, which is beneficial for ARV replication [49].

In addition to ARV, accumulating lines of evidence have suggested that several other viruses-induced autophagies play important roles in the viral life cycles and pathogenesis. Human tumor viruses (*γ*-herpesviruses) Epstein-Barr virus (EBV) and Kaposi's sarcoma-associated herpesvirus (KSHV), hepatitis C virus (HCV), rotavirus, and hepatitis B virus (HBV) have evolved various strategies to either subvert or enhance autophagy pathway for their replication and pathogenesis (Table 1).

In the course of ARV infection, activation of autophagy with rapamycin increased yield of ARV, while inhibition of the autophagosomal pathway by chloroquine treatment resulted in a decrease in virus production [47]. Consistent with this, suppression of autophagic proteins (Beclin-1, Atg7, and LC3) that are responsible for autophagosome formation by means of shRNA strategy or inhibitor (MA-3) led to a reduced viral transcription and/or ARV replication [9]. Furthermore, knockdown of expression of either LAMP2 or Rab7a, the two proteins required for the fusion of autophagosomes with lysosomes [50], significantly decreased virus replication; these results indicate that autophagy facilitates the propagation of ARV. A similar phenomenon has been observed with other viruses, such as hepatitis C virus [37] and rotavirus [39], and simian virus 40 (SV40) [41], where viral nonstructural proteins induced autophagy which in turn increased either viral replication or cell survival.

Rotavirus activates calcium-mediated signaling pathway and AMPK to trigger autophagy [39]. NSP4, a pore-forming protein (viroporin), activates a calcium signaling pathway by eliciting the release of calcium from the lumen of endoplasmic reticulum (ER) into the cytoplasm in the rotavirus infected cell. Consequently, the cytoplasmic calcium triggered a calcium signaling pathway involving calcium/calmodulin-dependent protein kinase kinase 2 (CAMKK2) and AMPK to activate autophagy that was then utilized for trafficking viral protein and facilitate virus replication. SV40 small T antigen also inactivates PP2A to activate AMPK that enables survival of cancer cells under glucose deprivation via the inhibition of protein synthesis and activation of autophagy as an alternative energy source [41]. Dreux et al. reported that accumulation of autophagic vesicles and conversion of endogenous LC3 to LC3 II were increased in HCV-infected cells [51]. Similarly the activation of autophagy machinery is required for distinct stages of HCV infection, such as delivery of incoming viral RNA to the translation apparatus, initiation of HCV replication, and viral spread, but not required once the infection is established [51]. Recently, several HCV proteins have been demonstrated to be responsible for induction of autophagy [37, 52]. HCV NS5A is sufficient to promote autophagosome formation by enhancing promoter activity and protein expression of Beclin-1 [52]. In addition to NS5A, NS4B can form a complex with Rab5 and Vps34 resulting in increased autophagic vesicle formation [37].

Previous study suggested that herpes simplex virus type 1 (HSV-1)-encoded ICP34.5 binds to the autophagy protein Beclin-1 and inhibits Beclin-1 [40]. Upon autophagy initiation, Beclin-1 forms an activating complex with the class III PI3 kinase Vps34 for vesicle nucleation [53], so when ICP34.5 binds Beclin-1, it inhibits cellular autophagy. It was reported that KSHV (a *γ*-herpesvirus) has evolved strategies to interfere with various aspects of autophagosome formation: KSHV counteracts autophagy initiation by encoding a viral Bcl-2 homolog that strongly binds to Beclin-1 and acts as a repressor of autophagic vesicle nucleation [54, 55]. In addition, another viral protein, vFLIP, blocks autophagosome formation by binding to Atg3, thereby preventing LC3 processing [56]. In contrast to KSHV, it was found that binding of HBx protein of HBV with Vps34 stimulates autophagy induction [42].

## 4. Reovirus and Apoptosis

Cell death has been generally classified into two groups, apoptosis and necrosis. However, Clarke classified PCD into four types [57]: type I cell death (also called classical apoptotic cell death), type II cell death (or autophagic cell death), type III cell death (also called necrosis-like programmed cell death) defined as nonlysosomal vesicle degradation, and the other type of PCD that is unfortunately poorly understood. In any case, type II cell death and type III cell death seem to operate in a caspase-independent manner [58].

Very recently, crosstalk between apoptosis and autophagy has been proposed [59, 60]. Beclin-1, the major protein responsible for autophagy, has been demonstrated to interact with a number of proteins; it is worth noting that, in addition to autophagic proteins, Bcl-2 (known as an antiapoptotic protein) binds with Beclin-1 to functionally antagonize Beclin-1-mediated autophagy [61, 62]. Another apoptosis related protein, caspase-8, also participates in autophagy: caspase-8 activation during apoptosis can cleave Beclin-1 and lead to a decrease in autophagy [63]. These findings indicate the possibility that viral protein(s) might modulate the switch between autophagy and apoptosis to facilitate their own replication.

The mechanisms of ARV-induced apoptosis and its signaling pathways in cultured cells have been extensively explored [64–69]. A previous study revealed that ARV infection upregulated p53 via multiple pathways including Src, Ras, and PKC *δ* [66]. Activation of p53 leads to induction of Bax and Bax translocation from cytosol to mitochondria at middle to late stage of ARV infection (after 18 hour post infection, hpi). Moreover, activation of caspases-9 and -3 was also detected in ARV-infected BHK-21 cells, indicating that ARV-induced apoptosis was accomplished by a caspase-dependent mechanism. Interestingly, significant syncytia formation in ARV-infected BHK-21 cells was observed undergoing apoptosis, establishing a correlation between ARV virus replication and apoptosis in cultured cells [64].

It has been demonstrated that ARV protein *σ*C serves as an apoptosis inducer [67]. Overexpression of ARV protein *σ*C resulted in the upregulation of p53 and triggered apoptosis. Further investigation demonstrated that ARV infection or in the presence of *σ*C by itself caused apoptosis mediated through the DNA damage signaling pathway, as evidenced by upregulation of two DNA-damage-responsive genes, DDIT-3 and GADD45*α* [68].

Interestingly, ARV proteins p17 and *σ*C responsible for induction of autophagy and apoptosis, respectively, colocalize well with the autophagy indicator LC3, although neither ARV protein p17 nor *σ*C physically interacted with LC3 [9]. A previous study indicated that ARV triggers Akt at early time of infection (2 hpi), resulting in delayed apoptosis [69]. Considering that ARV induces cell death in the middle to late stages of infection [69], it is possible that ARV triggers survival signaling that protects the host cells from caspase-dependent apoptosis and benefits the progression of viral infection at the early stage of infection. However, the precise mechanism that ARV participates to switch between autophagy and apoptosis requires further investigation.

The ability of MRV to activate apoptosis depends on the different serotypes of viruses introduced to a host cell. MRV type 3 has previously been shown to trigger apoptosis more strongly than MRV type 1, and this difference mapped to the viral gene segments S1 and M2 [70]. These gene segments encode capsid proteins sigma 1 and mu 1, respectively. Sigma 1 is the major viral attachment protein, but it has also been shown in MRV type 3 to trigger reovirus-induced apoptosis through its binding to sialic acid on cell lines, which leads to activation of NF-*κ*B [71]. Previous work has demonstrated that viral mu1 protein alone can stimulate apoptosis in transfected cell lines, with MRV type 3 mu1 eliciting more caspase-3 activation than MRV type 1 mu1 [72]. Danthi et al. have shown that certain mutations in mu 1 from MRV type 3 result in a great reduction in virus-induced apoptosis in cell lines at high multiplicities of infection (MOI), which stems from a failure to adequately activate the proapoptotic transcription factors NF-*κ*B and IFR-3 [73].

MRV-induced apoptosis in cell lines may also be enhanced by soluble tumor necrosis factor-related apoptosis inducing ligand (TRAIL), released by MRV infected cells, which binds to death receptors (DR) 4, and DR5 found on the cell surface [74]. DR4 and DR5 signaling in certain types of reovirus infected cells results in the activation of caspase-8, which then activates downstream caspases [75]. In some cells, reovirus-induced apoptosis is dependent on caspase-8, as caspase-8 inhibitors block apoptosis [74]. Reovirus infection also results in the activation of many apoptotic effectors, including other caspases, JNK and c-Jun, calpain, Bid cleavage, and Smac/DIABLO [76]. Whether and how MRV-induced apoptosis connects to autophagy at this stage remains largely unknown.

## 5. Conclusions and Discussion

Undoubtedly, there are multiple mechanisms that different viruses exploit to modulate autophagy in their host cells, presumably to their replicative advantage. Autophagy clearly plays an important role in ARV replication [9]. ARV protein p17 induces autophagy by its action on three different pathways: p17 activates two pathways AMPK and the tumor suppressor PTEN that in turn block the activation of mTORC1, the major inhibitor of autophagy [9]. Furthermore, p17 induces PKR and eIF2*α* phosphorylation that lead to host translation shutoff and also to activating autophagy [9]. In contrast to ARV, the viral protein responsible for modulation of autophagy in MRV remain unexplored.

Ideally, an oncolytic virus used in cancer therapy replicates well in order to spread to and kill tumor cells more efficiently, as has been suggested [35]. Autophagy seems to contribute to MRV replication, although its tie to viral replication has not been shown directly as it has been in ARV infection. MRV replicates better in cells that have intact PKR pathways [77], suggesting that autophagy may enhance MRV replication.

MRV induces both autophagy and apoptosis in tumour cells; what is not known is whether and to what extent autophagy is ultimately responsible for tumour death by reoviruses. In MRV-infected transformed cells, apoptosis also contributes to viral oncolytic activity [78]. In fact, MRV type 3 (Dearing) has been the preferred clinically used oncolytic mammalian reovirus, and it has been shown to induce apoptosis more robustly than MRV type 1 (Lang), though their replication rates are very similar [70]. This leads to the question of whether autophagy and apoptosis are induced by the same upstream signals in infected cells or by distinct mechanisms. Since different ARV proteins are responsible for activating autophagy and apoptosis, it may be that MRV also activates these pathways through separate mechanisms. It is possible that reovirus simply induces autophagy and apoptosis sequentially, but not hierarchically. The sequential activation is supported by previous findings [9, 47, 69]. If there is no hierarchy between autophagy and apoptosis, then as suggested by Maiura et al. inhibition of apoptosis might lead to either cell survival or necrosis (depending on the outcome of autophagy) without affecting autophagy, and inhibition of autophagy may likewise not affect apoptosis [60]. Berger and Danthi observed that MRV-infected L929 cells treated with caspase inhibitors do die, but by a receptor interacting protein 1 (RIP1) kinase pathway that results in a type of cell death known as necroptosis, the programmed necrosis triggered by death receptor signaling [79]. Cells undergoing necroptosis also undergo extensive autophagy, which some believe is the major mechanism for cell death by necroptosis [80]. Therefore, the caspase-independent cell death pathway activated in MRV-infected cells may in fact be mediated by virus-induced autophagy. It would likewise be very helpful to determine if inhibition of autophagy in MRV-infected cells has any effect on apoptosis.

It seems possible, though, that apoptosis induction may shift the cell away from autophagy and autophagy-induced death in reovirus infections. Caspase activation in cells undergoing autophagy has been shown to cleave Beclin-1, which can then no longer interact with autophagosomes, but instead the cleaved protein interacts with mitochondria, promoting apoptosis [10]. ARV infection and p17 transfection of immortalized chicken embryo fibroblast (DF-1) and Vero cells both lead to an increase in Beclin-1 by 24 hpi [9], however, whether that goes down during peak times of ARV-induced apoptosis, as well as whether the cleaved Beclin-1 product is detectable, has not been determined yet. It will be very interesting in the future to clarify the connections between autophagy and apoptosis in reovirus infections.

## Figures and Tables

**Table 1:** The target of viral proteins on modulators of autophagy.

Virus	Viral protein	Direct target	Downstream^#^	References
Hepatitis C virus	Nonstructural protein 4B (NS4B)	Rab5 (early endosome, complex to PI3K)	None	[37]

Hepatitis C virus	NS4B	Vps34 (PI3K complex)	PIP3/Akt	[37]

Rotavirus	NSP4	Calcium/calmodulin-dependent protein kinase kinase 2 (CAMKK2)	AMPK	[38]

Rotavirus	NSP4	5′ adenosine monophosphate-activated protein kinase (AMPK)	TSC1/2	[39]

Herpes simplex virus type 1 (HSV-1)	ICP34.5	Beclin-1	LC3-II	[40]

Simian virus 40	Small T antigen	AMPK	mTOR	[41]

ARV	p17	p53	PTEN	[9]

ARV	p17	AMPK	mTORC1	[9]

ARV	p17	PKR/eIF2*α*	Beclin-1	[9]

Hepatitis B virus	HBV X protein (HBx)	Phosphatidylinositol 3-kinase class III (PI3KC3)	mTOR	[42]

KSHV, HVS, and MCV*	viral FLIP	Atg3	LC3	[43–45]

CHIKV and SINV	NSP4	eIF2*α*	ATF-4	[46]

Note: *Kaposi's sarcoma-associated herpesvirus (KSHV), herpesvirus saimiri (HVS), molluscum contagiosum virus (MCV).

^#^Downstream effect on autophagy resulted from the interaction of viral proteins with their direct targets.
